# Minor Surgery

**Published:** 1863-02

**Authors:** 


*Minor Surgery.*—A threatened *furunculus* may be easily aborted by subcutaneous section with a fine knife. But one

In searching for *bullets* lodged deeply in the body or extremeties, the ingenious method suggested by M. Nelaton in examining the wound of Garibaldi is worth bearing in mind. A probe was so contrived as to bring away particles of the lead if met. An olive-shaped body made of unpolished china was fixed at the end of a common probe. The least contact of this with the lead left a stain which the fluids present would not obliterate. "With this instrument, Prof. Zanetti became so certain of the presence of the ball in Garibaldi's ancle that he forthwith extracted it."

Good strong *horsehair* is found to serve excellently well where fine *sutures* are required as in operations or wounds sbout the eyelids or scrotum. They are quite indestructible aperture need be made through the skin. Through this the indurated portion may be thoroughly transfixed and cut across in every direction. Or a second puncture may be made and another complete section of the diseased part made at right angles with the first. After the bleeding has ceased the skin should then be painted with collodion. The operation is almost painless and the cure about certain.

An enlarged *bursa* had best have its contents discharged by a fine trochar and canula, and afterwards be injected with a solution of Iodine in Iod. Pot. and water. Some little caution may be advisable in very large bursæ to avoid inflammation, but rarely is there any considerable local or general disturbance. A single operation generally is sufficient to the cure.

Among the vast number of methods resorted to, to abort threatening *whitlow* or *thecal abscess*, we have found none so uniformly successful as blistering the part with Cerat. Cantharid. The vesicant should extend some considerable distance beyond the point involved in inflammation, The blister alleviates the local pain so remarkably as usually to be very grateful to the patient, and rarely fails to arrest the progress of the disease. The free incision so commonly recommended is always very much dreaded by the patient, and is only occasionally successful in relieving the disease.

and unirritating. They maintain their elasticity better than the fine silver wire, and are more easily removed when wished. They will be found very advantageous in scalp wounds, where it may happen suture is unavoidable. But all sutures about the scalp should be avoided if possible, as they always endanger erysipelas.*

---

*Prof. Bedford's Principles of Obstetrics.*—As predicted in our notice of this invaluable work last year, it has speedily passed to its third edition. This is evidence of a practical sort to Prof. Bedford that his *labors* are appreciated by the profession. Both at home and abroad, this book stands, deservedly, at the head of all treatises on the subject. We again commend it to the libraries and perusal of all our readers.

* Scalp sutures occasioned the erysipelas, which made the trivial wounds wounds of Senator Sumner, an entering wedge of Secession.

